# Is TrpM5 a reliable marker for chemosensory cells? Multiple types of microvillous cells in the main olfactory epithelium of mice

**DOI:** 10.1186/1471-2202-9-115

**Published:** 2008-12-04

**Authors:** Anne Hansen, Thomas E Finger

**Affiliations:** 1 Rocky Mountain Taste and Smell Center, Dept. of Cell and Developmental Biology, School of Medicine, University of Colorado Denver, Aurora, CO 80045, USA

## Abstract

**Background:**

In the past, ciliated receptor neurons, basal cells, and supporting cells were considered the principal components of the main olfactory epithelium. Several studies reported the presence of microvillous cells but their function is unknown. A recent report showed cells in the main olfactory epithelium that express the transient receptor potential channel TrpM5 claiming that these cells are chemosensory and that TrpM5 is an intrinsic signaling component of mammalian chemosensory organs. We asked whether the TrpM5-positive cells in the olfactory epithelium are microvillous and whether they belong to a chemosensory system, i.e. are olfactory neurons or trigeminally-innervated solitary chemosensory cells.

**Results:**

We investigated the main olfactory epithelium of mice at the light and electron microscopic level and describe several subpopulations of microvillous cells. The ultrastructure of the microvillous cells reveals at least three morphologically different types two of which express the TrpM5 channel. None of these cells have an axon that projects to the olfactory bulb. Tests with a large panel of cell markers indicate that the TrpM5-positive cells are not sensory since they express neither neuronal markers nor are contacted by trigeminal nerve fibers.

**Conclusion:**

We conclude that TrpM5 is not a reliable marker for chemosensory cells. The TrpM5-positive cells of the olfactory epithelium are microvillous and may be chemoresponsive albeit not part of the sensory apparatus. Activity of these microvillous cells may however influence functionality of local elements of the olfactory system.

## Background

Traditionally, the main olfactory epithelium (MOE) of mammals was said to contain only basal cells, supporting cells, and ciliated olfactory receptor neurons (ORNs) that utilize OR-type receptor molecules and the canonical G-protein-coupled transduction pathway via Gαolf, adenylyl cyclase III (ACIII), and cAMP [1]. However, a review of the literature suggests that this conventional view is too simplistic, e.g. microvillous ORNs are present in the olfactory epithelium of fishes and in the vomeronasal organ of mammals. Also, microvillous cells have been reported for the MOE of some mammals including humans [2-5]. A study by Rowley et al. utilizing HRP tracing claimed that at least some microvillous cells project directly to the olfactory bulb [6]. Braun and Zimmermann [4], utilizing ecto-5'-nucleotidase as a marker, detected microvillous cells in the MOE and suggested a mechanosensory function for these elements. Carr et al. reported microvillous cells in rats and concluded that these cells were non-sensory cells [7]. Functional studies revealed that mice with a disrupted cAMP pathway of ciliated ORNs are still able to detect certain odorants and conspecific chemosignals [8,9] suggesting the presence of non-traditional transduction mechanisms. Interestingly, further studies on the transduction pathway(s) present in the olfactory epithelium of rodents led to the conclusion that some types of transient receptor channels, e.g. TrpC6 or TrpM5, are expressed in what appear to be microvillous cells in the olfactory epithelium [10,11]. Kaske et al. examined various tissues containing cells that express the TrpM5 channel and postulated that TrpM5 is a potential marker for chemosensory cells [12]. Lin et al. (companion paper, this issue) describe cells in the main olfactory epithelium that express the transient receptor channel TrpM5. These cells are microvillous, but vary in size and morphology.

The goal of this study was to further examine the microvillous cells in the olfactory epithelium at the light and electron microscopic level in order to ascertain whether they meet the criteria of sensory or non-sensory cells. If these cells are sensory cells they should either project an axon to the olfactory bulb as do ORNs, or they should form contacts with sensory nerve fibers like the solitary chemosensory cells or other types of sensory cells (e.g. hair cells, Merkel cells). We describe several types of microvillous cells, discuss their features, and conclude that the main olfactory epithelium of mice contains multiple subtypes of microvillous cells and that TrpM5 is not necessarily a marker for sensory cells.

## Methods

### Animals

Wild-type (C57BL/6) and TrpM5-GFP mice were bred in the animal facilities of the University of Colorado Denver, Medical School. TrpM5-GFP mice (kindly provided by Dr. Robert Margolskee, Mount Sinai School of Medicine, New York) contain a TrpM5-GFP construct including 11 kb of mouse TrpM5 5' flanking sequence, TrpM5 exon 1 (untranslated), intron 1, and the untranslated part of exon 2, and eGFP [13]. We used the polymerase chain reaction (PCR) to genotype the offspring for the presence of GFP. Animals were 1 to 6 months old. All procedures were in compliance with the University of Colorado Animal Care and Use Committee.

### Light microscopy

Mice were anesthetized with 20% chloral hydrate (2 mg/g body weight), perfused transcardially with 0.9% saline followed by 4% paraformaldehyde in 0.1 M phosphate buffer (PB). The olfactory organs were dissected and postfixed in the same fixative for 15 min to overnight. Cryoprotection was carried out in 30% sucrose overnight. Cryosections (12 – 14 μm) were mounted on Superfrost Plus slides (VWR, West Chester, PA) and frozen at -80°C until further use. Standard immunocytochemical procedures were used. Briefly, cryosections were rinsed in 0.1 M phosphate buffered saline (PBS), blocked in blocking solution containing 1% BSA, 3% normal donkey serum, and 0.3% Triton X-100 in PBS for 2 hours, and then incubated in the primary antisera overnight to 2 days. For details of antibodies see Table 1. After 3 washes, 20 min each, the sections were incubated in the appropriate secondary antibodies (Alexa 488, Alexa 568, 1:400; Invitrogen, Carlsbad, CA) for 2 hours at room temperature. After incubation, sections were washed 3 times 20 min and coverslipped with Fluormount-G (Fisher Biotech, Birmingham, AL). Control slides were treated either without the primary antibody or it was substituted by normal rabbit serum. Where available, the antisera were adsorbed with the appropriate peptides. Control sections showed no labeling. Sections were viewed under a fluorescence microscope or a confocal laser microscope (Olympus; Center Valley, PA).

**Table 1:** Antisera used to characterize TrpM5 cells

**Antisera against**	**Marker for**	**Company**	**Lot**
Calbindin	calcium-binding protein involved in calcium signaling	SWANT CB38	9.03
Calretinin	calcium-binding protein involved in calcium signaling	SWANT 7699/4	18299
CGRP	trigeminal nerve fibers	Peninsula Lab. T-4032	040826-4
Chromogranin A	neuroendocrine cells	Santa Cruz Sc-1488	D0507
CK18	supporting cells	Chemicon MAB3234	0507004430
Espin	actin-binding protein in microvilli	Dr. J. Bartles, Northwestern University, Chicago	-
GFP	green fluorescent protein	Abcam AB290	207431
Galphaq/11	G-protein subunit q/11/14 – transduction component	Santa Cruz Sc-392	F1107
IP3R3	IP3 receptor 3 – transduction component	Chemicon AB9076	25041643
Na^+^, K^+^-ATPase	sodium-potassium pump	Biogenesis 0126-2000	773/272892
NSE	neuronal and neuroendocrine cells	DAKO M0878	05437
PDE2A	transduction component	Santa Cruz Sc-17227	L0402
PGP 9.5	neuronal and neuroendocrine cells	AbD Serotec 7663-0504	071207
OMP	olfactory neurons	Dr. F. Margolis, University of Maryland	-
P2X2	purinergic receptor – transduction component	Alomone Labs APR003	AN-06
P2X3	purinergic receptor – transduction component	Chemicon AB5895	0602021455
PDE2A	transduction component	Santa Cruz Sc-17227	L0402
PGP 9.5	neuronal and neuroendocrine cells	AbD Serotec 7663-0504	071207
PLCbeta2	transduction component	Santa Cruz Sc-206	B0907
SNAP 23	synapses	SynapticSystems 111202	-
SNAP 25	synapses	Calbiochem NE1014	30933
Substance P	trigeminal nerve fibers	Accurate YMC1021	E9381
SUS-1	supporting cells in the main olfactory epithelium	gift of Dr. F. Margolis, University of Maryland	-
SV2	synapses and some nerve fibers	Developmental Hybridoma Bank	-
Synaptophysin	synapses	Epitomics 1485-1	YE269
Substance P	trigeminal nerve fibers	Accurate YMC1021	E9381
TrpC6	transient receptor potential channel C6 – transduction component	Abcam AB12249	342108
VAChT	"membrane transport protein"	Chemicon AB1578	24080681
Villin	actin-binding protein in microvilli	Beckman-Coulter 0258	1D2C3

### DiI tracing

Wild-type and TrpM5-GFP mice were perfused as described above. The heads were collected and fixed in 4% PFA overnight. Then the skull above the olfactory bulbs was opened. A small crystal of DiI (Molecular Probes; Eugene, OR) was placed into each olfactory bulb close to the cribriform plate by means of an insect pin. The skull was closed with a layer of 2% agar. Then the heads were placed in 4% PFA at room temperature for 3 to 4 weeks. After incubation the olfactory organs were dissected and embedded in 15% gelatin (Sigma; St. Louis, MO). 40 to 50 micron sections were cut on a vibratome (Ted Pella, Inc.; Redding, CA) and viewed under a fluorescence microscope or a confocal laser microscope (Olympus; Center Valley, PA). The DiI had traveled into all areas of the olfactory epithelium.

### Transmission electron microscopy

Wild-type mice: Mice were anesthetized and perfused with saline as before but the fixative was 4% glutaraldehyde in 0.1 M phosphate buffer. The olfactory organs were dissected and postfixed in the same fixative overnight. After rinsing in phosphate buffer, the tissue samples were postfixed with 1% osmium tetroxide for 2 h. The fixed specimens were dehydrated in a graded series of ethanol and acetone and embedded in Epon-Araldite (Electron Microscopy Sciences, Hatfield, PA). Ultrathin sections (silver to gold) were stained with uranyl acetate and lead citrate and examined with a FEI Tecnai G^2 ^electron microscope (Philips, Eindhoven, Netherlands).

Transgenic mice: For immunolabeling at the electron microscopic level, the animals were perfused, cryoprotected and processed as described for Light Microscopy with the following exceptions: 30 to 50 micron floating sections were cut and collected in 0.1 M PBS and treated with 3% H_2_O_2 _for 15 min. Triton X-100 was replaced with 1% saponin. Incubation times were extended to 3 days for primary antibodies and overnight for secondary antibodies. Secondary antibodies were biotinylated and visualized with standard ABC/DAB methods. After the DAB reaction the sections were washed in PB and postfixed in 4% glutaraldehyde overnight followed by a rinse in PB and postfixation in 1% osmium tetroxide for 1 h. Then the sections were treated as described for Electron Microscopy of wild-type mice and embedded between two Aclar sheets (Ted Pella; Redding, CA). Some of the ultrathin sections were viewed without uranyl acetate and lead citrate staining.

Figures were created in Adobe Photoshop, Version 7.1. In some micrographs dirt spots were removed with the clone stamp tool from areas where no tissue was involved.

## Results

**Figure 1:**
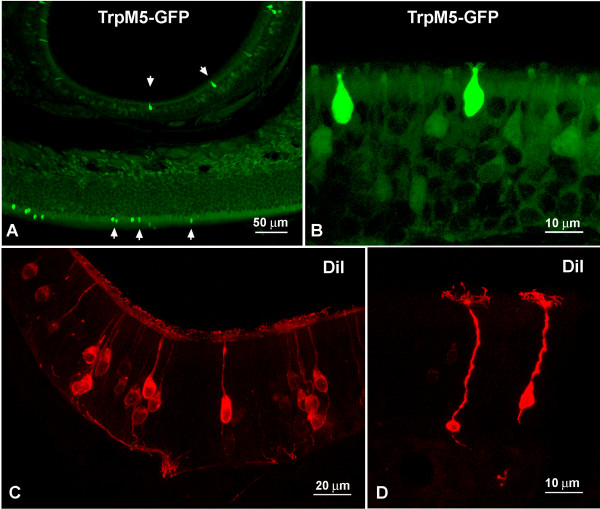
**Cell types in the MOE of mice**. A Different types of microvillous cells (MV) (arrows) in the MOE labeled by GFP. Note that the olfactory epithelium varies in thickness. B Higher magnification of TrpM5a MV cells. C ORNs retrogradely labeled from the olfactory bulb by DiI. D Typical long slender ciliated ORNs retrogradely labeled by DiI.

In transgenic mice where the TrpM5 promoter drives the expression of green fluorescent protein (GFP), GFP-positive cells are scattered throughout the main olfactory epithelium (Fig. 1). Lin et al. [14] focused on the TrpM5-positive ORN population whereas the current paper and a companion paper (Lin et al., this issue) examine non-ORN cell types.

Even at the light microscopic level, several morphological types of GFP-positive cells are obvious. The typical morphology of ORNs is evident in specimens backfilled with DiI from the cribriform plate. The ORNs are long slender bipolar cells with a thin dendrite bearing an olfactory knob with laterally radiating cilia (Fig. 1C, D). At the base of the ORN, an axon tapers towards the basal lamina (Fig. 1C, D) and aggregates with other ORN axons to form olfactory nerve fascicles. These fascicles penetrate the basal lamina and form the fila olfactoria. The majority of these TrpM5-GFP-positive ORNs lie in the ventrolateral zone of the MOE. Only a few, faint TrpM5-GFP-positive ORNs were present in other areas.

Non-ORN type GFP-positive cells are scattered throughout the main olfactory epithelium without an obvious pattern (Fig. 1A, B). These cells reach the surface of the epithelium but are shorter than ciliated ORNs; some span about half of the height of the OE, others span only the uppermost third (Fig. 1A, B). The upper portion of these cells is usually thicker than the dendrites of the typical ciliated ORNs. The apical appendages of these TrpM5-GFP-positive cells are shorter than the cilia of regular ORNs. The TrpM5-GFP label of these shorter cells is much brighter than that of the ORNs.

**Figure 2:**
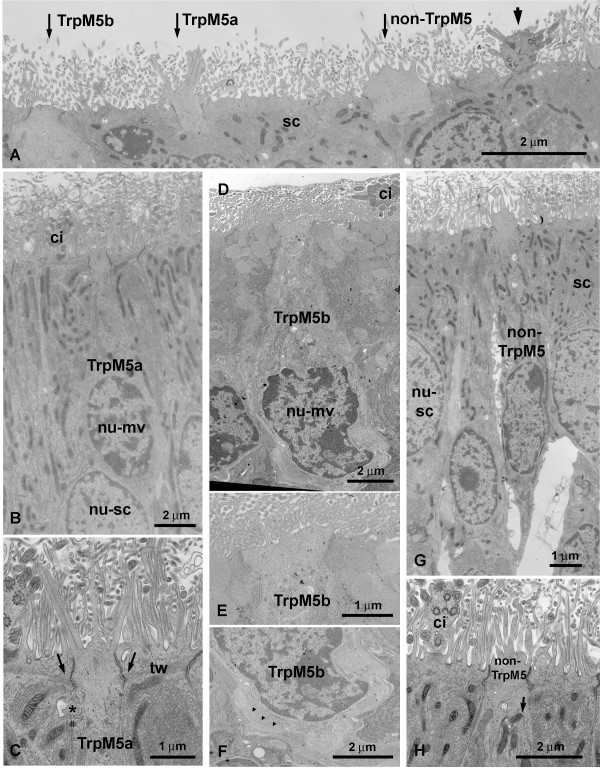
**Electron micrographs of different MV cells in the MOE of wild type mice**. A Apices of cells in the MOE. Thick arrow: ciliated ORN, thin arrows: different types of MV cells. B TrpM5a MV cell with "stiff" microvilli. nu-mv – nucleus of MV cell; nu-sc – nucleus of supporting cell (SC). C Higher magnification of apex of TrpM5a MV cell in B. tw: The terminal web does not continue in the TrpM5a MV cell (arrows). (*)"spine"; ci – ciliated ORN. D TrpM5b MV cell. ci – ciliated ORN; nu-mv – nucleus of MV cell. E Higher magnification of cell apex in D. F Higher magnification of basal part of TrpM5b MV cell. Small protrusions interdigitate with other cells (arrowheads). G A non-TrpM5 MV cell with a tapering basal part. nu-sc – nucleus of SC. H Higher magnification of a non-TrpM5 MV cell. Arrow – centriole with rootlet; ci – ciliated ORN.

Since the light microscopic level does not allow for detailed description of cell features we processed wild-type and TrpM5-GFP mouse OE for electron microscopy. These experiments revealed multiple microvillous cell types. As a preliminary classification we call these microvillous cells TrpM5a type, TrpM5b type, and a non-TrpM5 type on the basis of GFP-experiments and ultrastructural features. All 3 cell types occur in the main olfactory epithelium between ciliated ORNs and supporting cells (Fig 2A). The nuclei of the 3 microvillous cell types show a checkerboard pattern that typically has been used to distinguish ORN nuclei from the more homogenous nuclei of the supporting cells (Fig. 2B, D, G; 3C). In none of the 3 cell types described here did serial ultrathin sections reveal an axon penetrating the basal lamina. As our immunohistochemical experiments reveal (see below), the population of non-TrpM5 type microvillous cells consists most likely of several subsets with different molecular features, and it is possible that one or more of these subpopulations might have an axon that escaped our detection.

**Figure 3:**
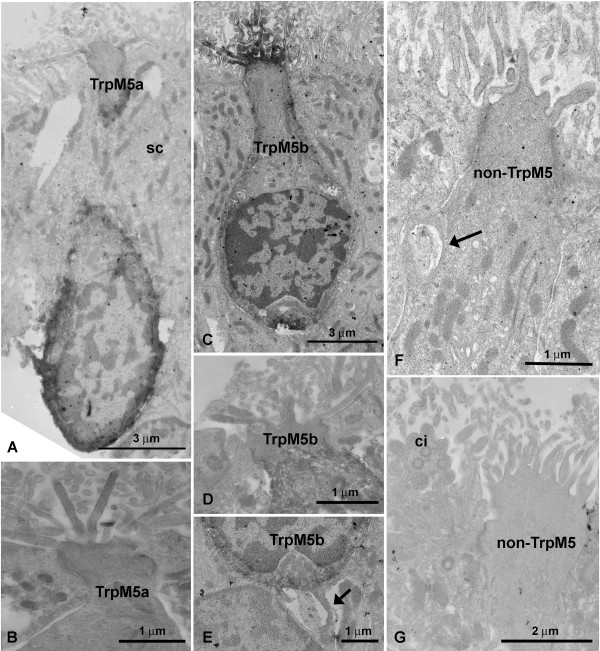
**Electron micrographs of different MV cells in the MOE of transgenic mice**. A TrpM5a MV cell labeled with the GFP antibody. Note how the neighboring SC "wraps" around the GFP-+ cell. B Higher magnification of a GFP-+ TrpM5a MV cell. The microvilli radiate from the apex giving the impression of being "stiff". C GFP-+ TrpM5b MV cell. Note the difference in cell size compared to the TrpM5a cell in A. D Higher magnification of the apex of a TrpM5b MV cell. E Basal portion of a TrpM5b MV cell. Small protrusions extend into the interstitium (arrow). F Non-TrpM5 MV cell in a TrpM5-GFP mouse. Arrow: nerve fiber profile adjacent to MV cell. G A non-TrpM5 MV cell in a TrpM5-GFP mouse with different morphology than that in F suggesting that non-TrpM5 MV cells comprise several subpopulations. ci – ciliated ORN.

### TrpM5a type microvillous cell

The TrpM5a type microvillous cell is a medium-sized microvillous cell spanning half of the height of the olfactory epithelium (ca. 20 micrometer) (Fig. 2B). The main difference compared to the other types of microvillous cells as seen in the electron microscope are the microvilli. TrpM5a type cell microvilli radiate straight from the cell apex giving the impression of being stiff (Fig. 2A, C). The apex of the cell is slightly elevated but does not form a knob as in microvillous olfactory receptor cells of fish. The microvilli are 150 – 180 nm wide, 2.0 – 2.5 micrometer long (Table 2), i.e. thicker than those of supporting cells but almost as long (Fig. 2C). The cell body contains abundant free ribosomes, some coated vesicles and some filaments, especially in the upper portion of the cell. Mitochondria are longitudinally arranged and more electron-lucent than those of non-TrpM5 type microvillous cells and supporting cells. The filaments of the terminal web of the supporting cells contact TrpM5a type microvillous cells but do not continue within the cell (Fig. 2C). Beneath the junctional complex spines reach out and often "dig" into adjoining supporting cells (Fig. 2C). Centrioles – typical for microvillous olfactory receptor cells are rare. The base of the TrpM5a type microvillous cell tapers out as seen in many non-TrpM5 type microvillous cells. In the TrpM5-GFP-transgenic mouse immunolabeling for GFP confirms expression in TrpM5a type microvillous cells implying that they normally express the TrpM5 channel (Fig. 3A, B).

**Table 2:** Characteristics of microvilli

	**Length**	**Width**
**Microvilli of TrpM5a cell**	2.0–2.5 μm	150–180 nm
**Microvilli of TrpM5b cell**	1.3–1.5 μm	110–130 nm
**Microvilli of non-TrpM5 cell**	1.2–1.8 μm	80–130 nm
**Microvilli of supporting cells**	2.0–2.6 μm	65–90 nm

### TrpM5b type microvillous cells

TrpM5b type microvillous cells only span the upper third of the olfactory epithelium and the length of their cell bodies ranges from 10 to 15 micrometer (Fig. 2D). Their cell bodies are pear-shaped and the lower half of the cell is filled with the nucleus. With 110 – 130 nm, the width of the microvilli of this cell type lies between those of TrpM5a type and non-TrpM5 type cells. Their length (1.3 – 1.5 micrometer, Table 2) is similar to the length of non-TrpM5 type microvillous cells and the shape of the microvilli is also similar to that of non-TrpM5 type cells, i.e. they do not give the impression of being stiff as seen in TrpM5a type microvillous cells. The apex is flat and not elevated as in TrpM5a type microvillous cells (Fig. 2E). Occasionally, the upper cell body contains multivesicular bodies and/or centrioles(s) (not shown). These centrioles are not connected to rootlets as seen in some non-TrpM5 type microvillous cells. Mitochondria are more electron-lucent and shorter than in TrpM5a type microvillous cells. Free ribosomes are less abundant. The basal part of TrpM5b type cells reveals several small filopodia-like appendages that often interdigitate with protrusions of neighboring cells (Fig. 3E). In transgenic TrpM5-GFP mice, TrpM5b type microvillous cells are GFP-positive indicating that they express the TrpM5 channel as do TrpM5a type microvillous cells (Fig. 3D, E).

### Non-TrpM5 type microvillous cells

The cell bodies of non-TrpM5 type microvillous cells have about the same length as those of TrpM5a type cells (ca. 20 micron) (Fig. 2G). The small microvilli occasionally branch (not shown). The width of these microvilli ranges from 80 to 130 nm, their length from 1.2 to 1.8 micrometer. Thus, they are thicker but shorter than the microvilli of supporting cells (65 – 90 nm width, 2.0 to 2.6 micrometer length, Table 2, Fig. 2G, H). The microvilli sprout from a small knob that is more pronounced than in TrpM5a type microvillous cells. Occasionally one or more centrioles are present in the upper part of the cell beneath the zonula occludens. Small rootlets may or may not be attached to these centrioles (Fig. 2H). Centrioles and rootlets are not present in all microvillous cells and the range of width and length of the microvilli is larger than in TrpM5a type and TrpM5b microvillous cells. Therefore it is possible that the cells described here as non-TrpM5 type microvillous cells represent more than one cell population (see below). The neighboring supporting cells contain a dense terminal web which contacts the microvillous cells. Contrary to the situation in TrpM5a type and TrpM5b microvillous cells (Fig. 2C, E), some filaments of the terminal web are also present in some non-TrpM5 type microvillous cell (Fig. 2H). The apical knob and the cell body contain few light vesicles and coated vesicles. The cell body contains several Golgi apparati, free ribosomes, and bundles of electron-dense filaments. The mitochondria are electron-dense (Fig. 2G, H) whereas the mitochondria in TrpM5a type and TrpM5b cells are electron-lucent (Fig. 2B, D). The nuclei are located at the level of the nuclei of supporting cells, sometimes even higher. The height of the cell from apex to the base where it tapers out is about 20 micrometer. Occasionally, non-TrpM5 type cells are in close contact with nerve fibers. Sometimes the cell "wraps" around these fibers (Fig. 3F). However, we did not detect obvious synapses. This microvillous cell type does not express GFP in the transgenic TrpM5-GFP mouse (Fig. 3F, G).

To directly test the possibility that these 3 cell types might have an axon, the fluorescent tracer DiI was placed into the olfactory bulb close to the cribriform plate. In TrpM5-GFP mice the green fluorescence was lost due to the long incubation in the fixative so that clear double-labeling with DiI was not possible. DiI traveled well into the OE, labeling the typical long slender ORNs. Only rarely was a medium-sized cell spanning the upper half of the epithelium labeled with DiI (Fig. 1C, D). Cells that span only the upper third of the epithelium, i.e. where the TrpM5a and TrpM5b microvillous cells are, were not labeled with DiI.

Given the fact that we could not detect an axon penetrating the basal lamina in either electron microscopic or DiI preparations, we conducted numerous immunohistochemical experiments to substantiate the notion that at least some of these microvillous cells are non-olfactory if not non-sensory cells. We utilized a panel of markers for various cell features (Table 1). Our experiments show that multiple subsets of TrpM5-GFP-negative microvillous cells exist. For the time being we classify these as non-TrpM5 type microvillous cells. Experiments to further distinguish the cell types classified here as non-TrpM5 type cells are underway.

**Figure 4:**
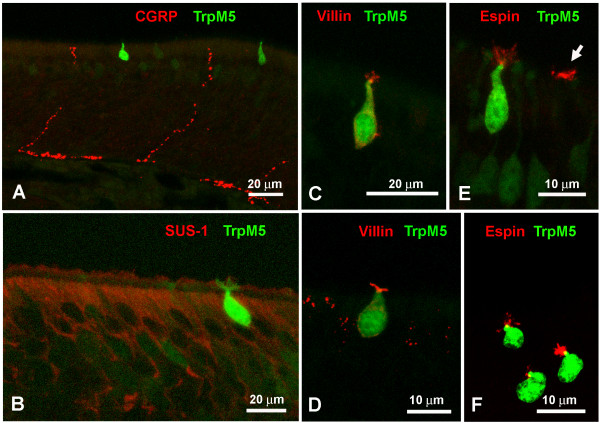
**A CGRP-positive nerve fibers are present but do not contact TrpM5-+ cells.** B SUS-1 labels SCs in the MOE but not TrpM5-+ cells. C GFP-+ TrpM5a cells show villin immunoreactivity in their microvilli and in their cell membranes. D Microvilli and the cell membrane of a GFP-+ TrpM5b cell labeled with villin antisera. E GFP-+ TrpM5a type cells show espin immunoreactivity in their microvilli. In addition to TrpM5-GFP-+ cells some non-TrpM5 cells show espin label (arrow). Some ORNs are slightly GFP-+ but are espin-negative. F Microvilli of GFP-+ TrpM5b cells labeled with espin antisera.

In order to test whether the microvillous cells are innervated by trigeminal nerve fibers [15], we used antisera against CGRP and Substance P, markers for trigeminal innervation. Nerve fibers expressing CGRP or Substance P were labeled within and below the olfactory epithelium (Fig. 4A) but we did not see any contact with the TrpM5-GFP-positive microvillous cells. However, it is possible that non-TrpM5 type cells are in contact with nerve fibers since we found microvillous cells at the electron microscopic level that are adjacent to nerve fibers (see Fig. 3F). Since the TrpM5-GFP cell bodies lie in the layer of the nuclei of the supporting cells, we tested the SUS-1 antibodies known to label supporting cells in the main olfactory epithelium [16]. TrpM5-GFP cells did not express SUS-1 (Fig. 4B) although surrounding supporting cells do.

Despite the fact that TrpM5a type and TrpM5b type microvillous cells clearly differ in morphology, their expression pattern of cell markers was identical (Table 3). Both types were labeled with espin and villin, markers for microvilli (Fig. 4C–F). In addition to the TrpM5-GFP-positive microvillous cells, some TrpM5-GFP-negative cells of microvillous morphology also were labeled with espin suggesting that these cells belong to the non-TrpM5 type microvillous cell group (Fig. 4E).

**Table 3:** Antibody pattern in microvillous cells

	**TrpM5a Type MV cells**	**TrpM5b Type MV cells**	**Non-TrpM5 Type MV cells**	**Ciliated ORNs**	**Other tissue controls**
TrpM5	+++	+++	-	+*	-
Calbindin	-	-	?	++	
Calretinin	-	-	?	++	
Chromogranin A	-	-	-	-	some cells in VNO
CK18	-	-	++	-	
Espin	+++	+++	+++	-	
CGRP	no contact	no contact	?	no contact	contact to SCCs
Gq/11	-	-	++	-	
IP3R3	-	-	+++	-	
Na^+^, K^+^-ATPase	-	-	-	-	nerve fibers, olfactory bulb
NSE	-	-	?	++	
OMP	-	-	?	+++	
P2X2	-	-	-	-	+ **
P2X3	-	-	+	+	
PDE2A	-	-	+	++***	
PGP9.5	-	-	?	+++	
PLC beta2	-	-	+	+	
Substance P	no contact	no contact	?	no contact	contact to SCCs
SNAP 23	-	-	?	-	synapses in muscle tissue
SNAP 25	-	-	?	-	synapses in muscle tissue
SUS-1	-	-	-	-	supporting cells
SV2	-	-	++	-	nerve fibers, synapses in various tissues
Synaptophysin	-	-	++	++	
TrpC6	-	-	++***	-	
VAChT	-	-	-	-	+ in SCCs
Villin	+++	+++	+++	-	

+++ = abundant; ++ = some; + = few; - = absent; ? = possible but not confirmed by electron microscopy

MV = microvillous cells

* weakly expressed in some ORNs in limited areas

** P2X2 positive cells are present in the thin epithelium of the turbinates which resemble solitary chemosensory cells (SCCs)

*** in limited areas

**Figure 5:**
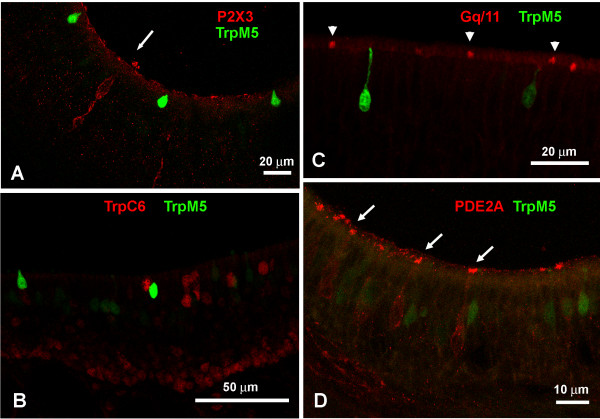
**A P2X3 receptor antiserum labels cells with nuclei high in the epithelium but does not label TrpM5-GFP-+ cells (arrow).** B Cells located high in the epithelium labeled for TrpC6 show no TrpM5-GFP label. C GFP-+ TrpM5a cells do not double-label with antisera against Gαq/11. Other presumably MV cells (non-TrpM5) are Gαq/11-+ (arrowheads). D GFP-+ TrpM5a cells do not express PDE2A. Other cells in the vicinity are PDE2A-+ (arrows).

To test whether the microvillous cells in question are olfactory neurons we applied OMP, PGP 9.5 and Hu-D, antisera that label ciliated ORNs. Neither TrpM5a type nor TrpM5b microvillous cells expressed OMP or PGP 9.5 (see Lin, companion paper, this issue), or Hu-D (data not shown) underlining their non-olfactory/non-neuronal character. In order to uncover possible features of these microvillous cells, we tested various antibodies against members of transduction pathways (e.g. P2X3 (Fig. 5A), TrpC6 (Fig. 5B), Gαolf, Gαq/11 (Fig. 5C), PDE2A (Fig. 5D)), synaptic components (e.g. SV2, synaptophysin) and other common cell markers (e.g. CK18, Na+, K+-ATPase, data not shown). Other than espin and villin none of the cell markers used in this study labeled the TrpM5-GFP cells categorized as TrpM5a type and TrpM5b microvillous cells. However, several of these markers were expressed in cells with microvillous morphology (Fig. 5A–D) that were TrpM5-GFP-negative. These cells probably belong to the non-TrpM5 type group of microvillous cells and are presently investigated in a separate study.

## Discussion

Our study revealed at least 3 types of microvillous cells in the main olfactory epithelium of mice. Villin and espin, actin-binding proteins typically expressed in microvilli of chemosensory and mechanosensory systems [17] were the only cell markers that labeled all microvillous cell types, confirming that they all bear microvilli. Two of the microvillous cell types show the transient receptor channel TrpM5-GFP label (TrpM5a type and TrpM5b microvillous cells). In addition, electron microscopic as well as immunohistochemical experiments revealed microvillous cells that were TrpM5-GFP-negative and expressed other cell markers not seen in the TrpM5-GFP cells. We group these cells as non-TrpM5 type microvillous cells. None of the types of microvillous cells described in this study have an axon that penetrates the basal lamina nor did we detect synapses consistent with findings from other studies.

Kaske et al. studied cells in various tissues that expressed TrpM5 and postulated that TrpM5 is an intrinsic signaling component that plays a physiological role in olfaction of odorants and pheromones [12]. As TrpM5 is also present in cells of the taste and gastrointestinal system the authors claim that TrpM5 is a marker for chemosensory cells. Lin et al. also found solitary chemosensory cells in the respiratory epithelium that are TrpM5-positive [18]. If TrpM5 is a marker for chemosensory cells, the TrpM5-positive cells should express other characteristics of chemosensory cells including contact with the nervous system as well as members of the chemosensory transduction pathway, and/or other markers of ORNs like OMP, PGP 9.5 etc. In the case of the solitary chemosensory cells, Lin et al. showed that TrpM5-positive cells are also labeled by antisera against PLCβ2 and PGP 9.5, known markers for chemosensory cells [18]. Yet, none of these components double-labeled the microvillous TrpM5-GFP-positive cells in our study. Also, the antibody SUS-1 which is specific for olfactory supporting cells did not label the TrpM5-positive cells. Thus, based on the present study, we conclude that the TrpM5-GFP-positive cells – termed here TrpM5a type and TrpM5b type microvillous cells – are not among the cells traditionally described as comprising the olfactory epithelium, i.e. olfactory receptor cells, supporting cells, and basal cells. Since none of the neuronal cell markers tested showed a positive result, and the cells have neither an axon projecting to the olfactory bulb nor contact to nerve fibers of trigeminal origin, it is not even clear whether these cells are sensory cells at all. At least for chemosensory cells we question the reliability of TrpM5 as a marker. However, we do not rule out that the TrpM5-GFP-positive microvillous cells may be chemoresponsive and influence local mucosal elements through the release of diffusible mediators.

A recent study on mouse and rat olfactory epithelium described microvillous cells labeled with antibodies against IP3R3 and PLCβ2 [10]. These microvillous cells did not degenerate after bulbectomy suggesting that they are not ORNs. The authors claimed that these cells express the transient receptor channel TrpC6 although they did not show double-label experiments for TrpC6 and either IP3R3 or PLCβ2. In the present study we did not see any cells double-labeled for TrpM5 and TrpC6. Neither did the TrpM5-GFP-positive cells double-label for IP3R3 or PLCβ2 (see also Lin et al., companion paper, this issue). Consequently, we postulate that the TrpM5-GFP-positive microvillous cells are not the ones shown in the study by Elsaesser [10]. It is likely that the TrpC6 cells belong to the group of non-TrpM5 type microvillous cells.

In the past, several studies mentioned microvillous cells in the main olfactory epithelium of rodents, e.g. [19,20]. Since the morphological descriptions varied and the proposed functions were often controversial, these studies received little attention. Okano et al. [21] postulated that microvillous cells in the olfactory epithelium of dogs were precursors of supporting cells. Bannister [22], based on the electron microscopic descriptions of the chicken olfactory epithelium [23], suggested that microvillous cells may be precursors for ciliated olfactory neurons. This hypothesis was rejected by Jourdan [20] since both cell types have very distinct features and no "intermediate" stages between microvillous and ciliated olfactory neurons have ever been detected.

Others have attributed various functions to these microvillous cells in addition to the notion of being precursor cells. One central question was: Are the microvillous cells true ORNs? Rowley and coworkers conducted experiments with HRP as a neural tracer and showed that microvillous cells could be retrogradely labeled from the rat olfactory bulb [24]. The authors mentioned, however, that a small percentage of microvillous cells "did not effectively transport HRP" from the olfactory bulb to their somata. There is also the possibility of nonspecific labeling. A study on the OE of fish reported a third type of ORN labeled by fluorogold [25]. Later it was shown that the fluorogold labeled cells in the OE nonspecifically [26]. In the present study we did not find any evidence of an axon projecting towards the olfactory bulb which would have been proof that these microvillous cells are, indeed, ORNs. Although we cannot rule out completely that we may have missed cells with an axon in our ultrathin (70 nm) serial sections, we conclude that the microvillous cells described in this study are not ORNs.

Several authors reported microvillous cells without an axon, i.e. cells that were not ORNs. For instance, Carr et al. [7] described a microvillous cell in the rat olfactory epithelium. These cells had no axon and survived ablation of the olfactory bulb but did not react with the marker for supporting cells SUS-1. Thus these cells do not belong to the olfactory system in the strict sense of basal, supporting, and olfactory receptor cells.

Occasionally the morphological features of microvillous cells reported in the past are similar to the features seen in microvillous cells of this study. Miller and coworkers described microvillous cells in the rat olfactory epithelium in a study of regeneration following exposure to toxic compounds [27]. These cells had no synapses but finger-like processes extending between the supporting cells. Also, histochemical staining with ecto-5'-nucleotidase revealed microvillous cells in the rat olfactory epithelium that had numerous invaginations interdigitating with the cell membranes of neighboring supporting cells [28]. We noted numerous processes of TrpM5b and non-TrpM5 type microvillous cells interdigitating with other cells, and also invaginations in non-TrpM5 microvillous cells. Asan and Drenckhahn [5] described two types of microvillous cells in rat and mouse. Among others, the authors showed microvillous cells labeled with antibodies against villin and CK 18. In the present study we detected villin in TrpM5a, TrpM5b and non-TrpM5 microvillous cells whereas CK 18 labeled only non-TrpM5 microvillous cells. It is probable that some of the microvillous cells seen in this study may be the microvillous cells described by [7,27,4], or [5].

## Conclusion

Our study shows that the mouse olfactory epithelium contains different types of microvillous cells. The cell types described in this study do not have an axon, i.e. are not ORNs. The broad reaction spectrum to various cell markers seen in this study and in the literature implies the existence of several subpopulations of microvillous cells. Their function is enigmatic. Further experiments are needed to elucidate the role of these cell populations and their possible connection to the olfactory and/or other sensory systems.

## Authors' contributions

AH participated in the design of this study, carried out the experiments at the light and electron microscopic level, and drafted the manuscript. TEF participated in the design and coordination and helped with the manuscript. Both authors read and approved the final manuscript.
